# Enhancing onchocerciasis elimination program management: A biological approach to deciding when to begin Stop Mass Drug Administration activities

**DOI:** 10.1371/journal.pntd.0011348

**Published:** 2023-07-13

**Authors:** Daniel Boakye, Charles Mackenzie, Jamie Tallant, Anne Heggen, Sashi Leff, Lokemla Nadjilar, Moussa Sow

**Affiliations:** 1 Reaching the Last Mile Fund, The END Fund, New York, New York, United States of America; 2 Noguchi Memorial Institute for Medical Research, University of Ghana, Accra, Ghana; 3 NTD Support Center, Task Force for Global Health, Decatur, Georgia, United States of America; 4 Onchocerciasis Elimination Program, Ministry of Health, Republic of Tchad; 5 Onchocerciasis Elimination Program, Ministry of Health, Republic of Mali; Imperial College London, Faculty of Medicine, School of Public Health, UNITED KINGDOM

## Abstract

Understanding when it is the appropriate time to stop administering the drugs in a chemotherapy-centered treatment program such as onchocerciasis remains a challenge due to cost, imperfect testing procedures, and a lack of long-term experience. Different approaches for assessing when a program can begin the extensive stop-treatment surveys have been recommended, and tested, with varying results. We describe here a practical approach that is based on information on both transmission as well as infection. This new protocol first defines operational transmission zones (OTZs) based on vector breeding sites followed by an epidemiological assessment of the resident populations adjacent to these breeding sites. Basing decisions to stop MDA treatment based on breeding site locations (i.e., transmission zones) rather than on political administrative units, is a practical, cost-effective approach. Importantly, this biology-based approach is more closely related to the actual state of onchocerciasis transmission.

## Introduction

Eliminating onchocerciasis from endemic countries currently require at least 10 to 12 years of annual distribution of the anthelminthic ivermectin [1], and, in some cases, a longer period may be needed. National programs distribute the drug to the eligible community residents in the endemic areas through a procedure known as mass drug administration (MDA), i.e., treatment of everyone above 5 years who is not pregnant or infirm [2]. It is currently difficult for a program to define whether the level of transmission of *Onchocerca volvulus* (Ov) has reduced to a point where MDA can be safely stopped as the World Health Organization (WHO) defined criterion for elimination has been reached [3]. Various protocols aimed at assisting countries to assess this vital programmatic point have been developed over the past 10 or so years.

In 2010, the African Program for Onchocerciasis Control (APOC), in their conceptual framework for the elimination of onchocerciasis [4], recommended there be 2 steps in the decision to stop ivermectin treatment. Firstly, there should be an “epidemiological survey,” where approximately 10 high-risk communities are assessed to demonstrate that infection levels are close to, or have reached, levels that indicate that interruption of transmission has been achieved. This would then be followed by a more extensive phase of both epidemiological and entomological evaluations that lead to the all-important decision to stop treatment. In 2016, WHO published guidelines for the verification of onchocerciasis elimination [3] that recommended the omission of the initial, smaller scale, “epidemiological” survey, and that the Stop-MDA assessment should consist only of a combined epidemiological (Ov16 serology) and entomological assessment (O-150 Pool-screening PCR). They recommended that if these 2 parameters successfully demonstrated the interruption of transmission, then MDA with ivermectin could be stopped.

Then in 2017, the WHO Onchocerciasis Technical Advisory Sub-committee (OTS), a group established to provide additional guidance on the implementation of the 2016 WHO guidelines [5], proposed the carrying out of a Pre-Stop MDA evaluation employing Ov16 serology—a procedure similar to that proposed in the first phase of the 2010 APOC proposal. However, in addition, the OTS advised that 100 children aged between 5 and 9 years in 3 to 5 first-line villages per evaluation unit (EU) should be tested using a laboratory based Ov16 rapid diagnostic test. An “EU” was only loosely defined by the OTS in their advice.

## Towards a more biologically appropriate approach

It is common in NTD programs to use administrative units (e.g., official district boundaries) for the various management and monitoring steps. However, onchocerciasis lends itself to a more biologically nuanced approach. There is well-defined close relationship between the breeding sites of the transmitting vector for onchocerciasis, *Simulium* sp., with increased levels of infection in those people living near these breeding sites [6]. This biological fact is central to the argument that the optimal assessment unit, often called the “evaluation unit” (EU), be based on the presence of active breeding sites rather than governmental administrative boundaries. Developing a protocol to assess the impact of MDA based on this concept, and specifically deciding when to stop treatment, requires a detailed understanding of the bio-geographical aspects of *O*. *volvulus* transmission and the identification of the transmission zones for infection. To accomplish this in a programmatically practical manner, we have modified the original pre-stop approaches with a new procedure that defined areas as operational transmission zones (OTZs) and have tested it in the field in the END Fund’s Reaching the Last Mile Fund supported countries in Africa, namely in Mali and Tchad.

When country program teams are defining OTZs, they must be aware of the local onchocerciasis history and environmental characteristics. Biological onchocerciasis transmission zones can be quite varied in size across the different global endemic areas, with some being small enough to be used as the whole EU such as in the Americas or in the *S*. *neavei* areas in Africa. However, in many endemic countries in Africa, the biological transmission zones can be very large, especially if one takes into consideration the migratory range of the black fly vector species involved. In addition, these often cross national borders adding a complication to program management. Thus, to make the protocol practical, we have developed and applied the concept of an OTZ to identify areas within a country that can be assessed more rapidly to assist programmatic decisions. An OTZ is demarcated within a country based on hydrology (e.g., river basins), on the ecological conditions, on breeding site maps, as well as vector species composition and prevalence maps of *O*. *volvulus* infection. This approach does not use official administrative boundaries as these are unlikely to be related in any way to vector habits. A program’s EU is therefore an OTZ and not, as often before, an administrative or politically based district.

There are several factors that assist in defining an OTZ, which is essentially is an activity driven by an understanding of the local environment. Knowledge of the major rivers, disease prevalence, vector species distribution, and various environmental factors that drive the presence and clustering of breeding sites is essential, as is an understanding of the discontinuities related to these diverse factors. Defining an OTZ can be described as bringing commonalities together into environmental units related to the potential breeding of black flies.

**Fig 1:**
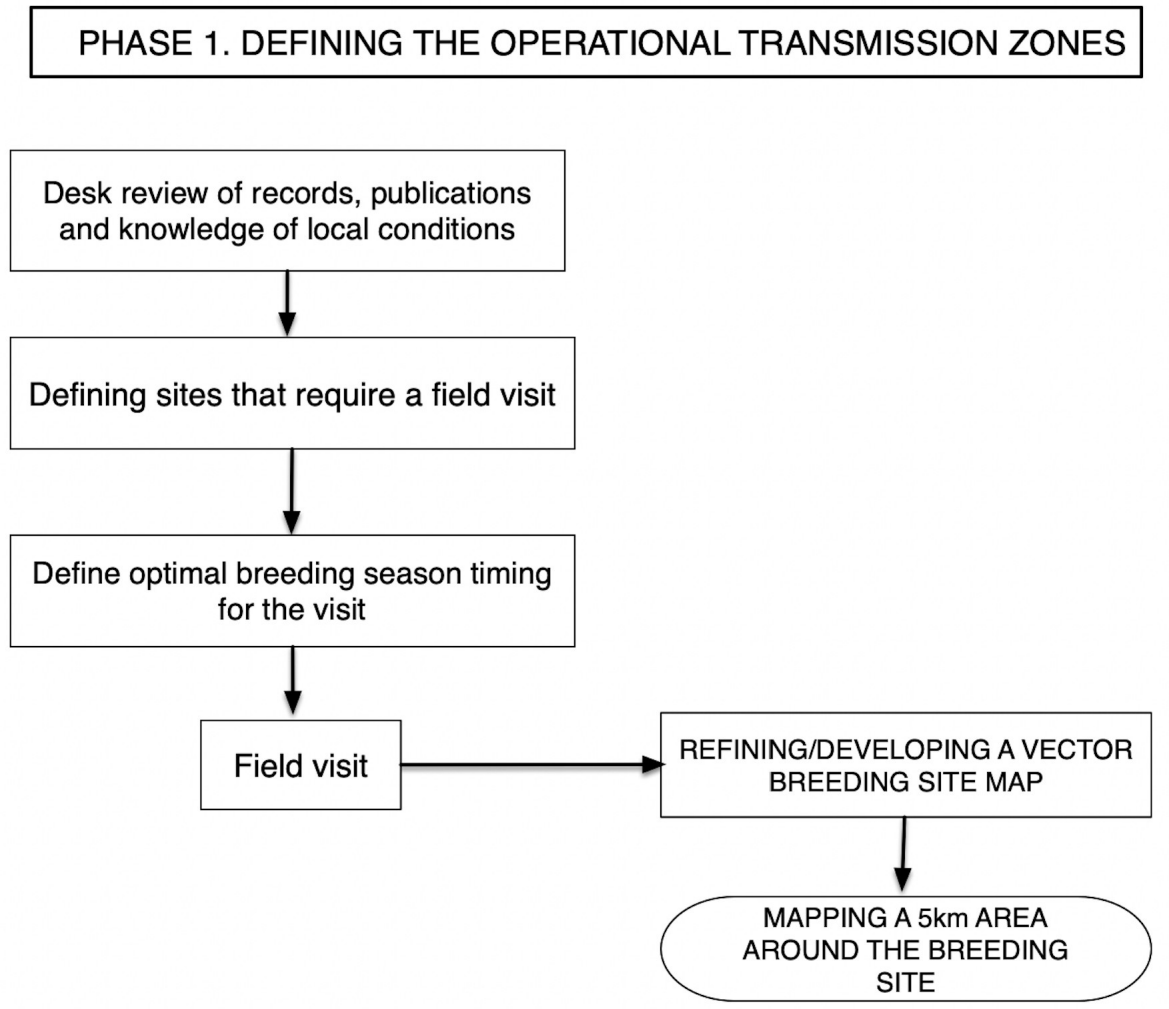
Pre-stop MDA survey Phase 1—Defining the OTZs. MDA, mass drug administration; OTZ, operational transmission zone.

**Fig 2:**
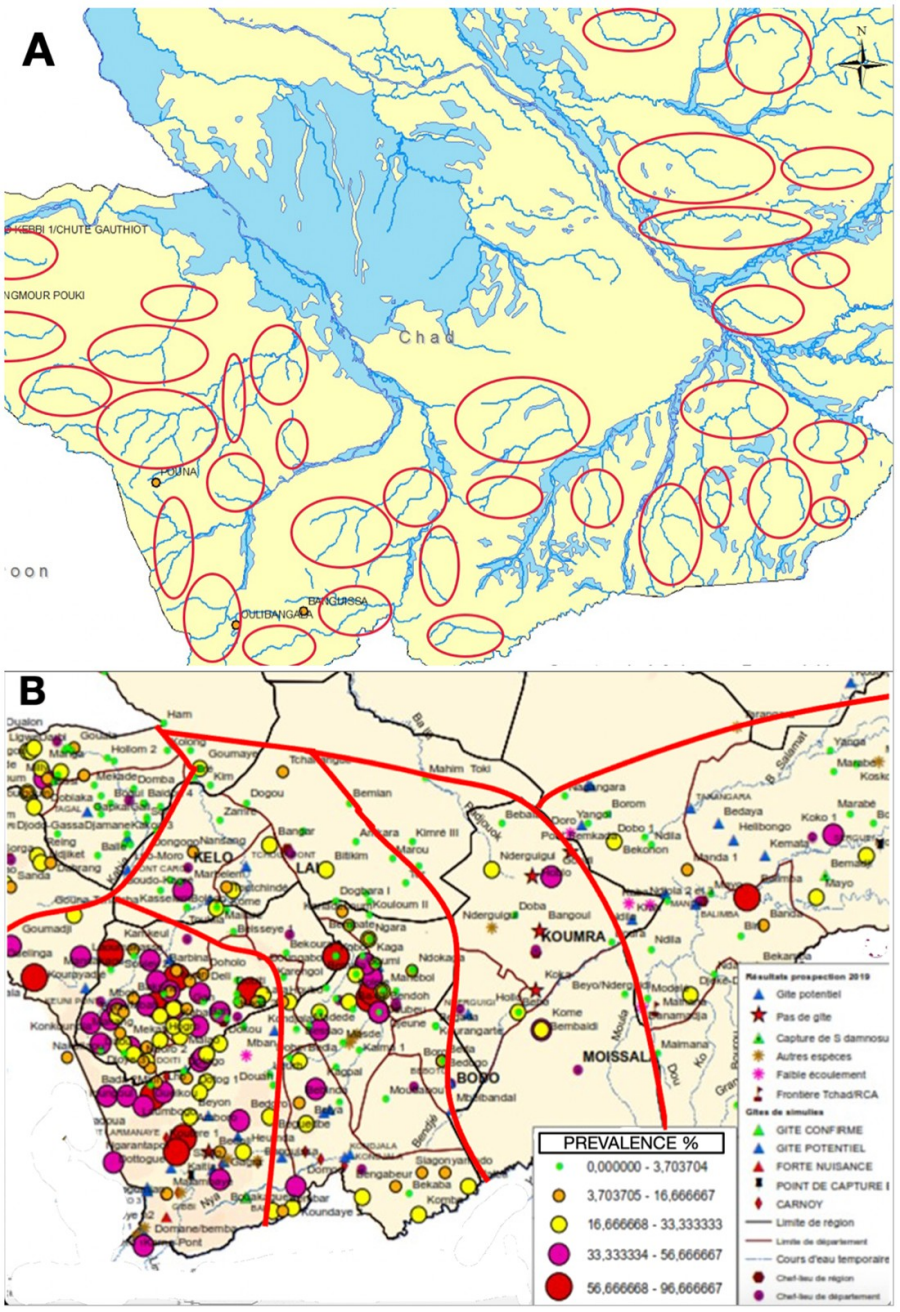
An example of establishing OTZs in Tchad. (A) Identifying potential breeding sites. A map of breeding sites that require assessment developed from a desk assessment of information available—red circles represent a possible potential or active onchocerciasis breeding site areas (35 in total). (B) Defining the OTZ. Following the assessment of the breeding sites, further consideration of the historical and environmental information, 5 OTZ were defined (red lines). OTZ, operational transmission zone.

The Pre-Stop MDA protocol we recommend is divided into 2 phases (S1 and S2 Figs). Phase 1 involves the updating (or creation) of the national map of breeding sites of the vector *Simulium* sp. in all the endemic areas (Figs 1 and 2A). Once “ground truthing” (i.e., through field visits) of potential breeding sites has been carried out to provide confirmatory information, and all the important environmental and historical information has been carefully assessed, OTZ are drawn (Fig 2B) to include all areas with onchocerciasis transmission potential in the country. Thus, an OTZ is a geographical area where transmission of Ov occurs by locally breeding vectors and that can be regarded as a natural ecological and epidemiological unit for programmatic interventions.

Understanding the local situation regarding any black fly breeding, any capability for supporting breeding, and any interactions between humans, can only be achieved by visiting the sites in question—commonly known as prospection (“ground truthing”). Factors such as the time of year, climate change, and the investigating team’s security all need to be considered. It may be necessary to revisit a site if the rainy season is delayed; however, careful planning of such prospection visits is valuable and can assist in making the time needed for such events brief and manageable.

**Fig 3:**
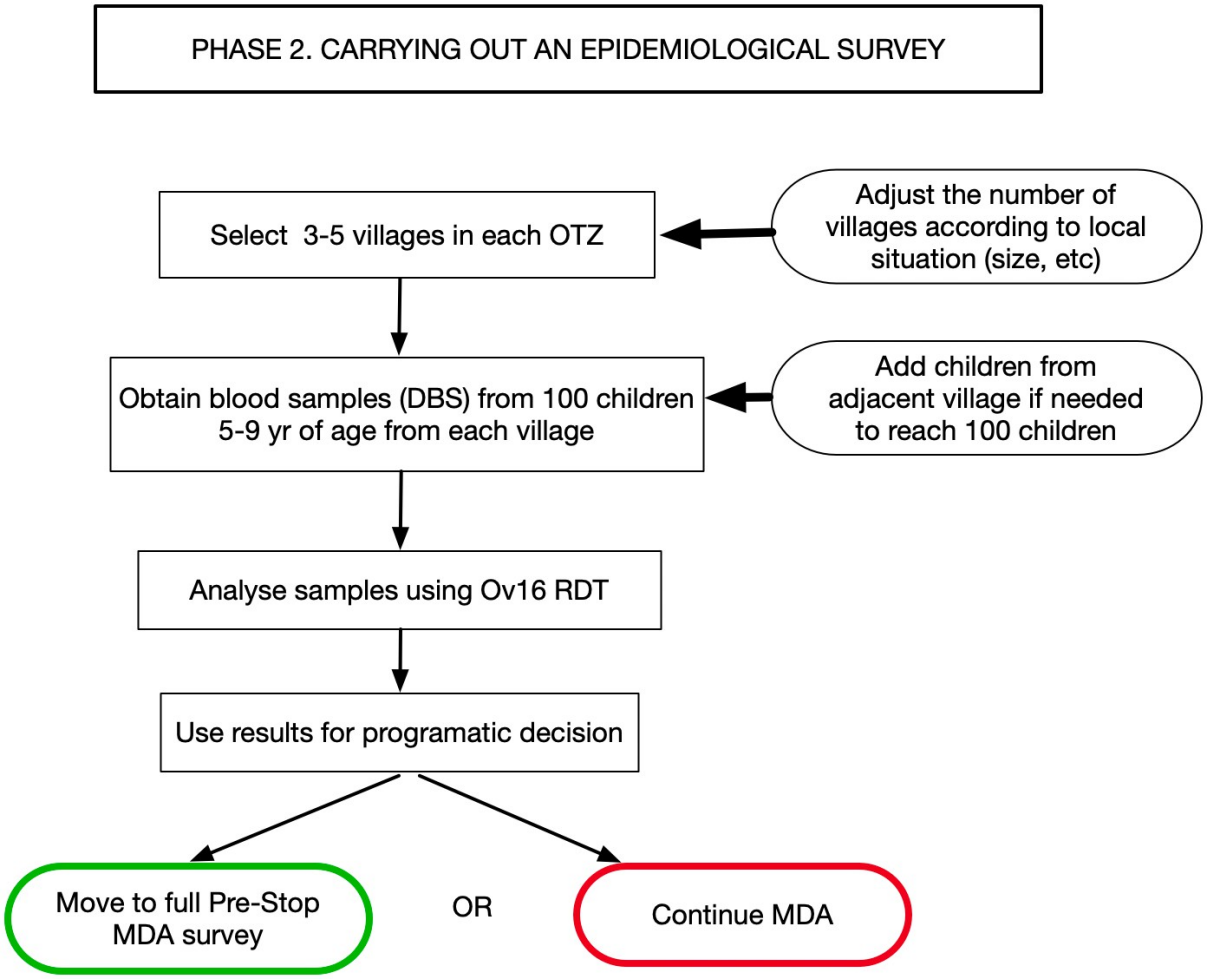
Pre-stop MDA survey Phase 2—Carrying out an epidemiological survey. MDA, mass drug administration.

Phase 2 is an epidemiological survey, using Ov16 serology of villages adjacent to every identified potential *Simulium* sp., breeding site with these OTZ (Fig 3). It should be noted that this pre-stop MDA process does not lead to a decision to stop MDA; it is a procedure aimed to show if a full Stop MDA survey, a much more expensive and time consuming activity, should be undertaken.

Thus, with the national OTZs defined, and with the knowledge of the breeding sites within each of the OTZs, the updated breeding site map is then used to select appropriate first-line villages for the next step, the epidemiological evaluation. The OTS recommended 3 to 5 villages per an “evaluation unit,” and our experience suggests that 3 to 5 villages are appropriate for most locations. At least 1 village within 5 km of each breeding site should be sampled in an OTZ; however, where several breeding sites are close together, 1 or 2 villages are enough to represent these breeding sites as the vectors are being sampled within the same area. An overall principle is that the total number of villages sampled in an OTZ should be representative of the OTZ in terms of size.

The evaluation villages in a Pre-Stop MDA should all be first-line villages and thus be within a 5-km zone around each breeding site present in the OTZ. However, it must be emphasized that the number of first-line villages selected per OTZ should be based on the size of the area and a consideration of any recent epidemiological or demographical information, e.g., the local conditions might indicate that assessing more than 5 villages is more appropriate. One hundred children, aged 5 to 9, are examined in each village; when there are less than 100 children present in a selected village, children living in very close villages are included to reach the required total. Finger prick samples are taken from these children using the dried blood spot (DBS) technique; these samples being collected and stored in the field and then transported and following current recommendations, subsequently processed in a laboratory for the presence of antibodies to *O*. *volvulus* using the Ov16 RDT [6]. As recommended by WHO, a prevalence of less than 2% in any OTZ indicates that this area can move to a full Stop MDA survey, and a prevalence above this indicates that MDA should continue.

## Advantages of this new approach

It is important that national onchocerciasis elimination programs have a manageable and practical means of gaining insight into the status of their ongoing MDA activities, i.e., a procedure that can be carried out with the least possible burden and cost to the elimination team. National programs need to know when the degree of transmission occurring in their MDA areas has reached the point where carrying out a full Stop MDA survey is likely to be successful. This Pre-Stop MDA procedure presented here is important as the currently recommended procedure to define the transmission status of a program, the full Stop MDA survey, is expensive and can be time consuming [6].

The approach we recommend here was developed using information and experience gained in onchocerciasis endemic areas in Africa. The protocol was found to be comparatively easy to implement and was cost-effective. As it uses river basins rather than official administrative units (e.g., Districts), to determine the first-line villages, it is more easily adapted to different countries irrespective of the administrative divisions used in a country. It should be emphasized that through this new approach, all endemic and potentially endemic areas are included in the defining of OTZs, and that an OTZ often borders another OTZ, e.g., where 2 river basins come together. In addition, an OTZ may in some cases cross national borders into another country. Further application of this new approach in different countries will support any needed modifications to this protocol and develop ways to apply this approach to any specific questions that may arise in each country. Many countries in Africa have reached the point in their onchocerciasis elimination programs where they soon will be needing to decide whether to consider stopping MDA.

The WHO OTS recommendation for Pre-Stop MDA evaluations is unclear as to what should be defined as an EU [7]. The use of EUs based on the identification of OTZ provides information more closely aligned to the actual disease epidemiology, and at a lower cost, than the information generated when ADs are used. We predict from our current experience in Tchad and Mali that using the OTZ-based pre-stop protocol we describe here, i.e., based on OTZ rather than AD, can reduce the number of areas needing evaluation by approximately a third in many endemic countries. Similarly, the adjustment of the number of first-line villages accounting for size and geographical distribution of breeding sites of a particular EU reflects the local situation more closely than does the OTS protocol. The OTS procedure of sampling first-line villages is important and valid, and, consequently, it requires solid knowledge of vector breeding sites. Thus, both pre-stop procedures require careful updating of breeding site maps as the first step, and knowledge of the river basins and ecological characteristics is essential to defining the EUs.

Our redefined Pre-Stop MDA protocol has been used successfully in Mali and Tchad. The use of an OTZ reflects the epidemiology of the disease and will likely reduce the cost of evaluations compared with when administrative districts or subdistricts are used as the EUs. In addition, there will always be the need to consider local issues and special circumstances that need to be taken into consideration. For example, it will be important to adjust of the number of villages selected for epidemiological sampling to ensure that it is truly representative of the area under evaluation. Consideration of the local situation will always be needed in decisions made by program managers.

In our view, the approach we describe here, with its increased emphasis on the biology of transmission, is a more suitable approach for defining when a program has reached the point when it should carry out the extensive full Stop MDA survey. This initial step, that is much less burdensome than the full survey, can give a country program increased confidence in their eventual decision to enact the full Stop MDA survey, and, then if the latter is successful, move towards a safe stopping of MDA activities.

It is important to emphasize that an OTZ here in this new protocol is an operational zone based on transmission biology and does not need to include the whole biological transmission zone. This use of the OTZ as the management unit is both operationally easier to manage, as well as being more biologically realistic as it is based on tenets of biology rather than nonbiological definitions such as political administrative districts. Although identifying breeding sites can be quite costly to a program, once completed, the information can be used for other stages in the pathway to elimination (OEM, Stop-MDA, and Post-MDA surveillance). It should be noted that the prospection segment of this procedure can be “decentralized” and all carried out, following appropriate training, at the District level by local personnel.

## Supporting information

S1 Fig Phases of the Pre-Stop MDA evaluation. (TIFF)

S2 Fig Phase 1. Determining where to visit.
(TIFF)

S3 Fig Phase 1. Determining when to visit.
(TIFF)

S4 Fig Phase 1. Field work.
(TIFF)

S5 Fig Phase 2. Epidemiological surveys; planning and field work.
(TIFF)

S6 Fig Phase 2. Epidemiological surveys; laboratory work and analysis of results.
(TIFF)
