# Supplementary figures and images for: Enhancing onchocerciasis elimination program management: A biological approach to deciding when to begin Stop Mass Drug Administration activities
Source: PLoS Negl Trop Dis. 2023 Jul 13;17(7):e0011348. doi: 10.1371/journal.pntd.0011348 (PMC10343055; doi:10.1371/journal.pntd.0011348)

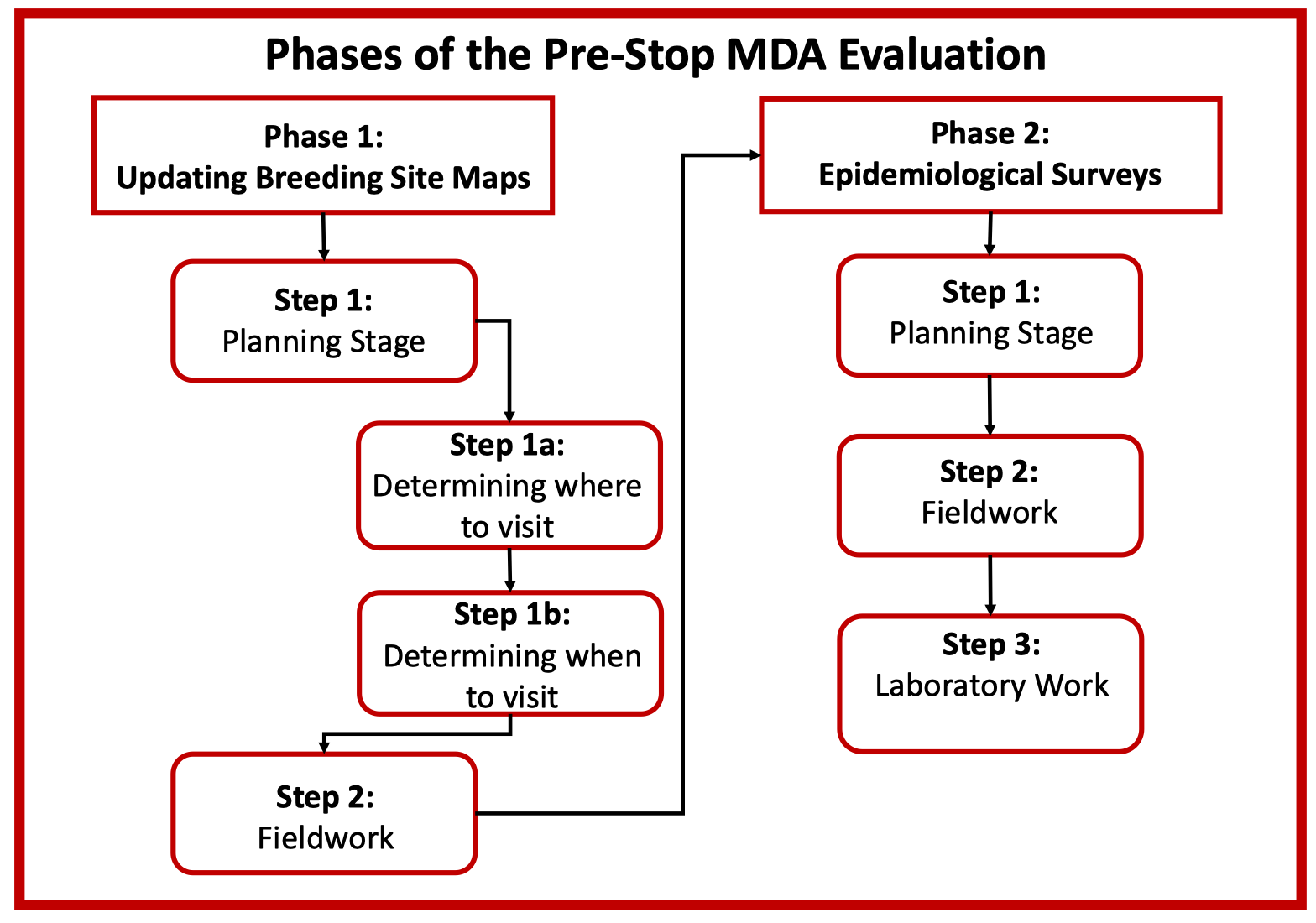

Supplement: S1 Fig — (TIFF) [file pntd.0011348.s001.tiff]

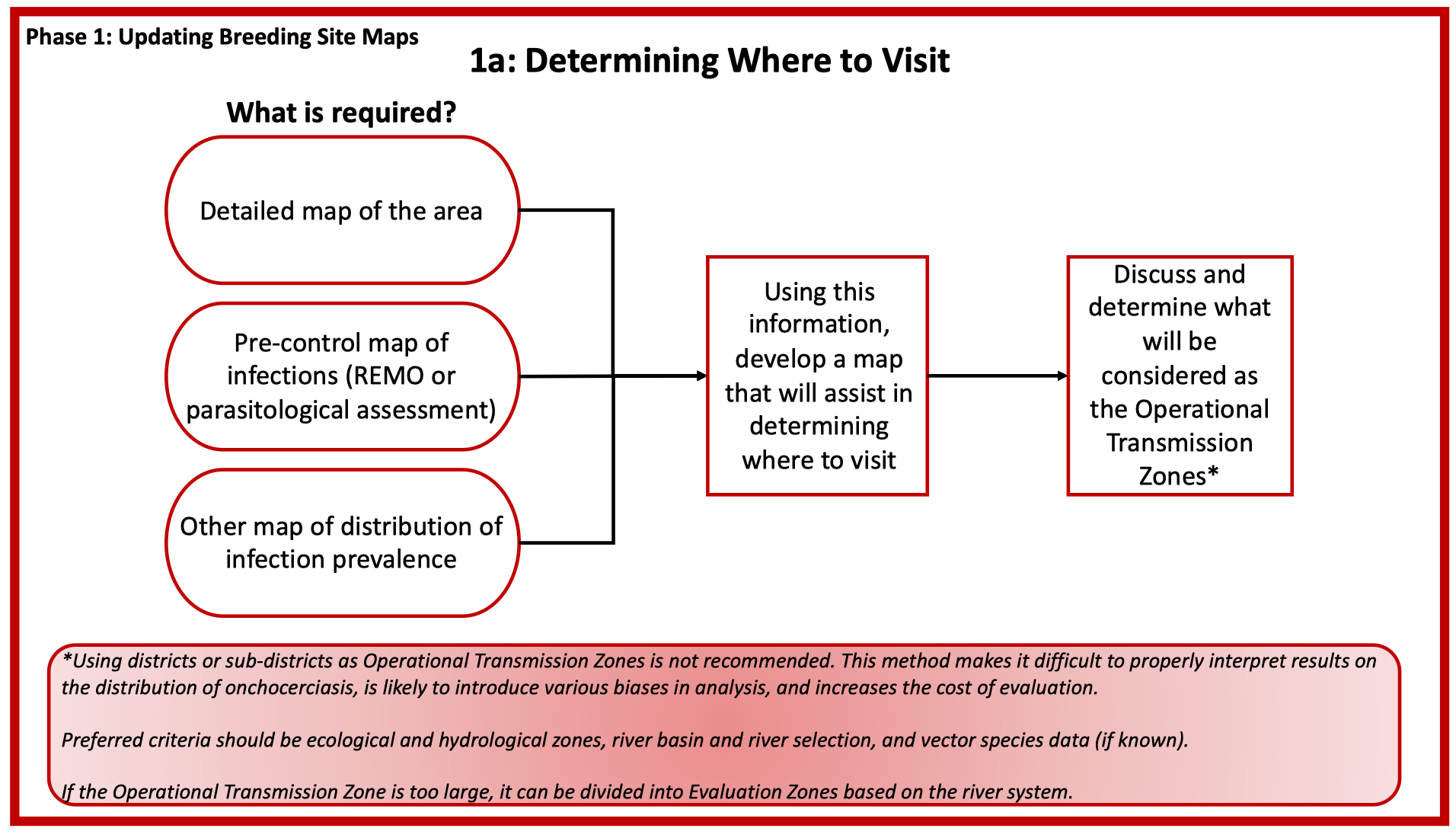

Supplement: S2 Fig — Determining where to visit. (TIFF) [file pntd.0011348.s002.tiff]

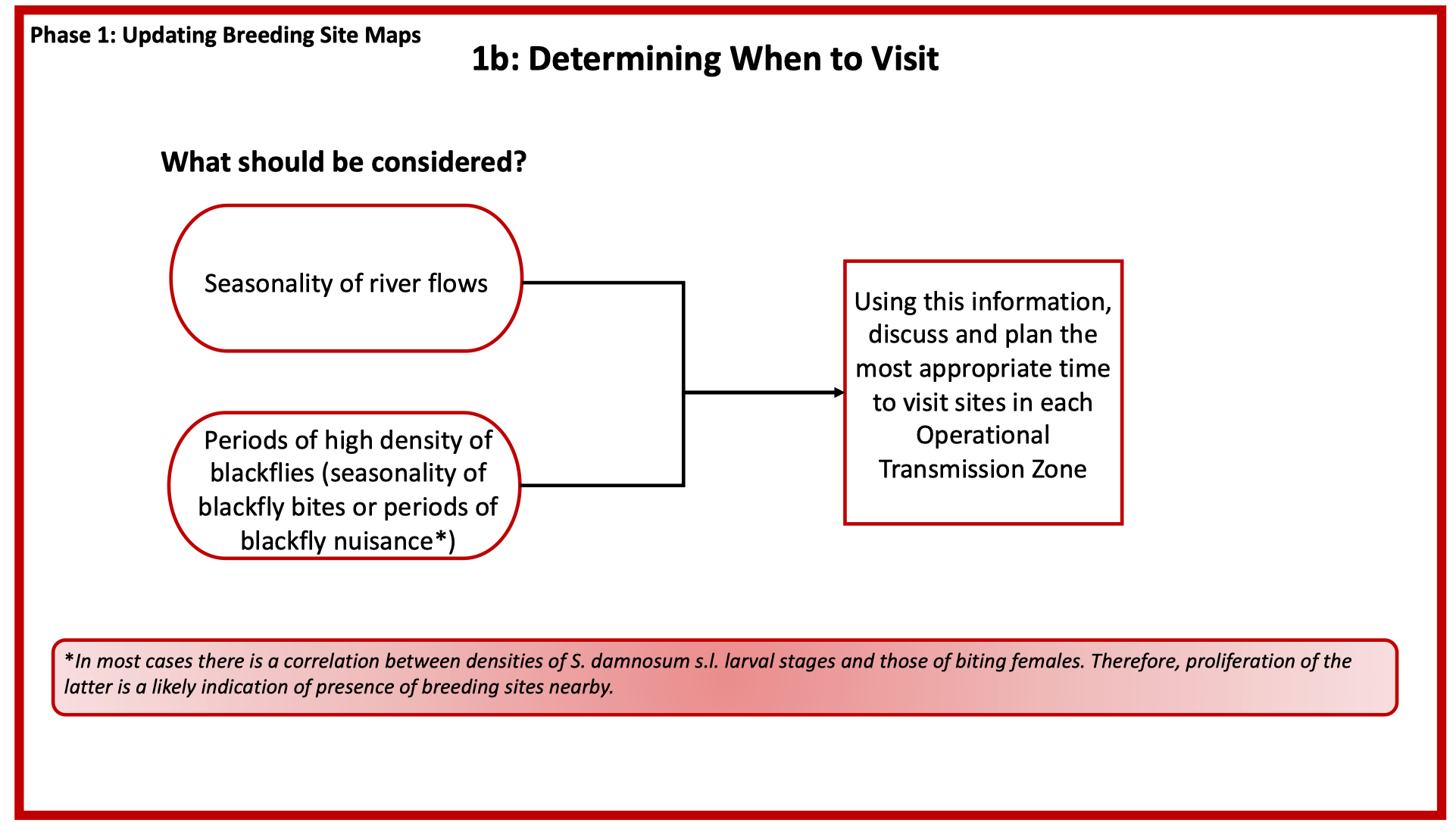

Supplement: S3 Fig — Determining when to visit. (TIFF) [file pntd.0011348.s003.tiff]

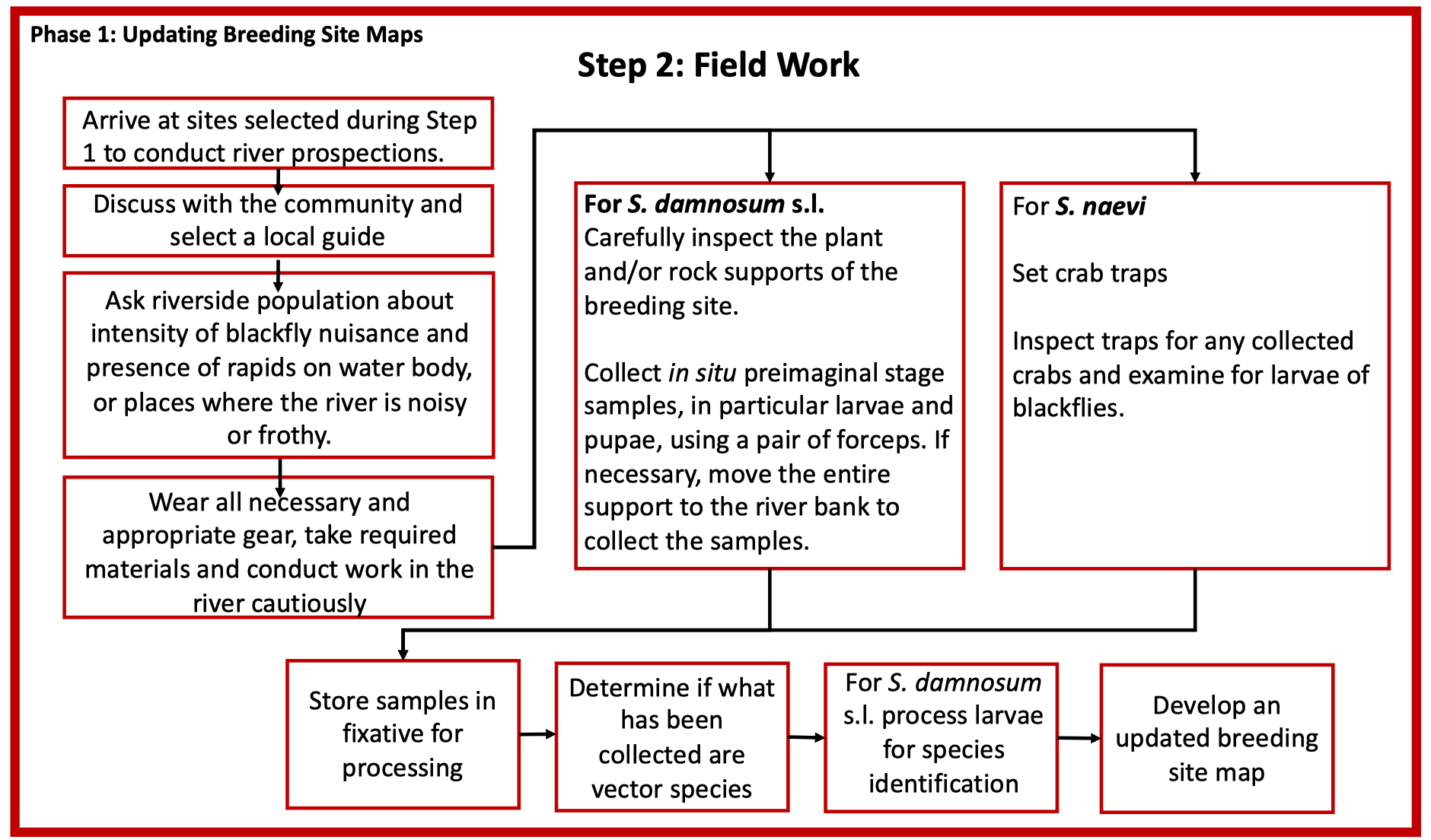

Supplement: S4 Fig — Field work. (TIFF) [file pntd.0011348.s004.tiff]

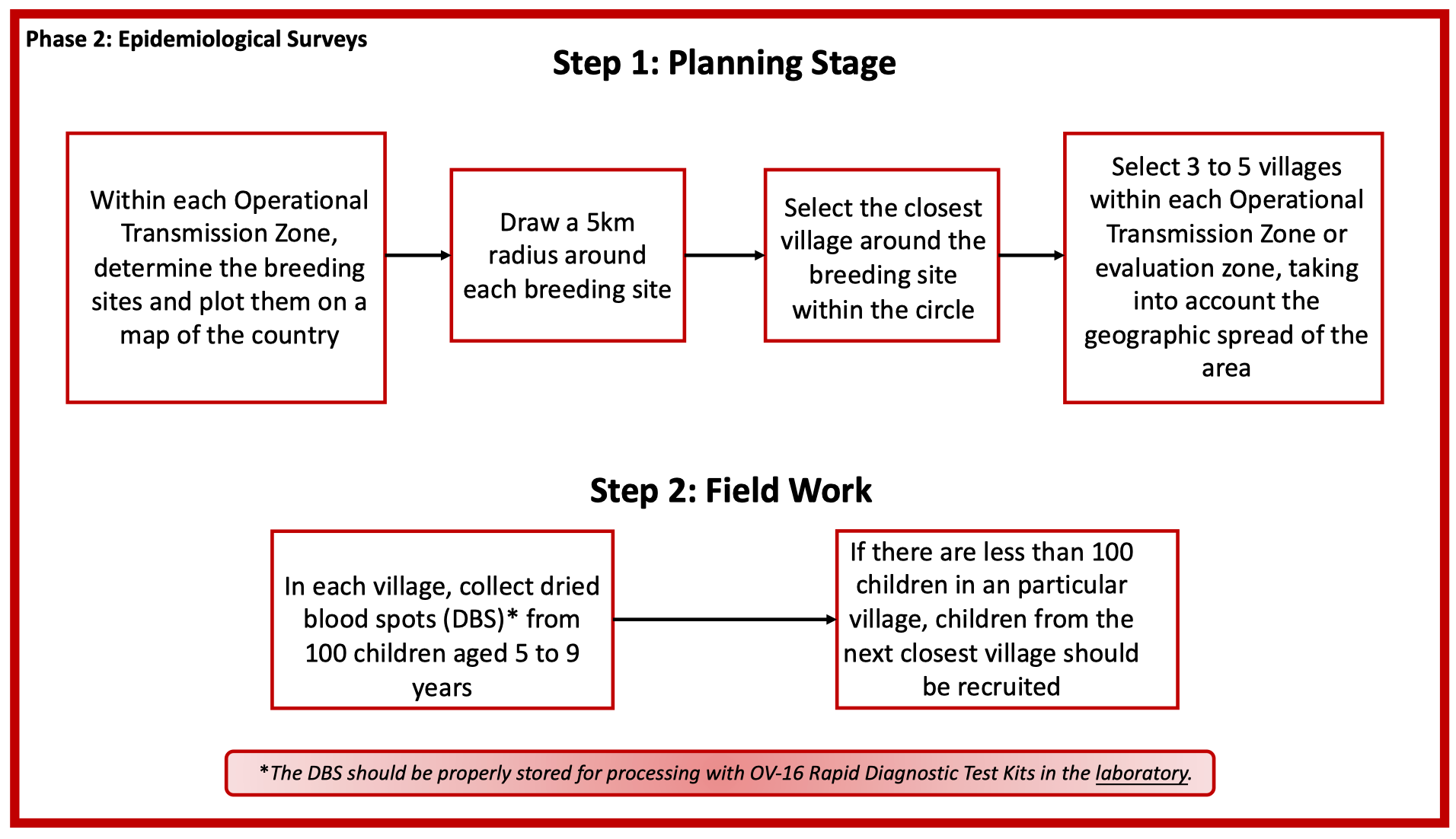

Supplement: S5 Fig — Epidemiological surveys; planning and field work. (TIFF) [file pntd.0011348.s005.tiff]

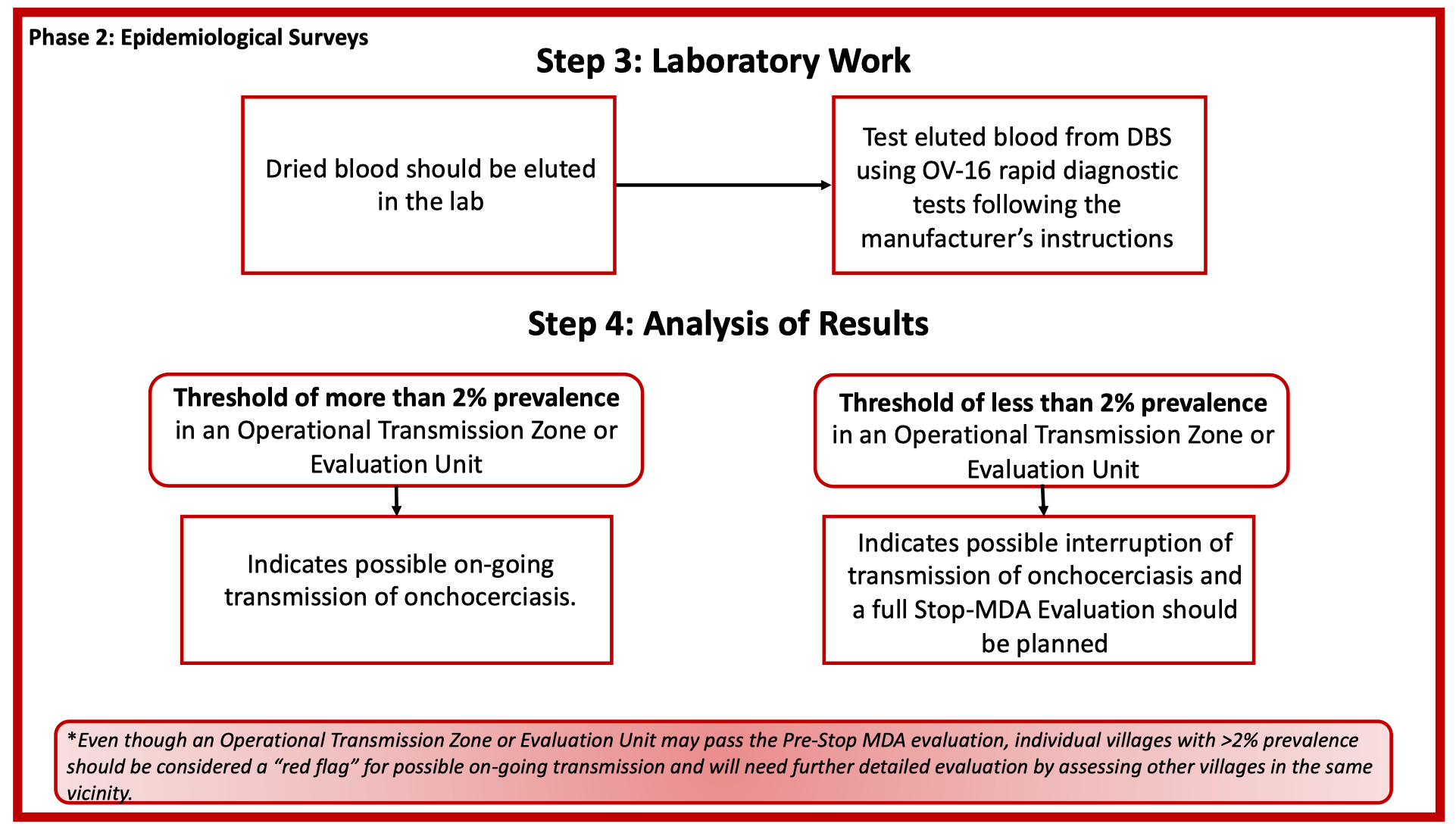

Supplement: S6 Fig — Epidemiological surveys; laboratory work and analysis of results. (TIFF) [file pntd.0011348.s006.tiff]
